# Social prescribing during the COVID-19 pandemic: a qualitative study of service providers’ and clients’ experiences

**DOI:** 10.1186/s12913-022-07616-z

**Published:** 2022-02-25

**Authors:** Stephanie L. Morris, Kate Gibson, Josephine M. Wildman, Bethan Griffith, Suzanne Moffatt, Tessa M. Pollard

**Affiliations:** 1 Department of Anthropology, Durham University, Dawson Building, South Road, Durham, DH1 3LE UK; 2 Population Health Sciences Institute, Faculty of Medical Sciences, Ridley 1, Newcastle University, Newcastle Upon Tyne, NE1 7RU UK

**Keywords:** Social prescribing, Behaviour/lifestyle interventions, Chronic/long-term conditions, Community interventions, Complex interventions, Patient engagement, COVID-19, Social determinants of health

## Abstract

**Background:**

COVID-19 public health restrictions, such as social distancing and self-isolation, have been particularly challenging for vulnerable people with health conditions and/or complex social needs. Link worker social prescribing is widespread in the UK and elsewhere and is regarded as having the potential to provide support to vulnerable people during the pandemic. This qualitative study explores accounts of how an existing social prescribing service adapted to meet clients’ needs in the first wave of the pandemic, and of how clients experienced these changes.

**Methods:**

Data were collected in a deprived urban area of North East England via remote interviews with clients (*n* = 44), link workers (*n* = 5) and service provider managerial staff (*n* = 8) from May–September 2020. Thematic data analysis was conducted.

**Results:**

The research found that service providers quickly adapted to remote intervention delivery aiming to serve existing clients and other vulnerable groups. Service providers experienced improved access to some existing clients via telephone in the first months of remote delivery and in some cases were able to engage clients who had previously not attended appointments at GP surgeries. However, link workers also experienced challenges in building rapport with clients, engaging clients with the aims of the intervention and providing a service to digitally excluded people. Limited link worker capacity meant clients experienced variable contact with link workers with only some experiencing consistent support that was highly valued for helping to manage their conditions and mental wellbeing. Limited access to linked services also adversely affected clients. Clients living in less affluent circumstances and/or with worse health were more likely to experience negative impacts on their long-term condition. Some found their health and progress with social prescribing was ‘on hold’ or ‘going backwards’, which sometimes negatively affected their health.

**Conclusions:**

Social prescribing offered valued support to some during the pandemic, but remote support sometimes had limited impact for clients and findings highlight the vulnerability of social prescribing’s success when linked services are disrupted. Findings also show the need for more to be done in the upscaling of social prescribing to provide support to digitally excluded populations.

What is known about this topic and what this paper adds What is known:

- During the COVID-19 pandemic many people have suffered hardship
- Social prescribing is being rolled out across the UK to support people with long-term needs
- A consistent, trusting and positive relationship with a link worker is central to successful social prescribing

What this paper adds:

- During COVID-19, this social prescribing service was able to adapt quickly to remote delivery but faced challenges, including accessing digitally excluded clients
- Some clients received greatly appreciated support while others felt that their social prescribing journey was ‘on hold’ or ‘going backwards’
- Link workers’ abilities to detect changes in people’s lives were paramount to successes of social prescribing during the COVID-19 pandemic

## Background

People with long-term conditions (LTCs), particularly multi-morbidities, tend to experience more severe illness and are at a higher risk of mortality from COVID-19 [1, 2]. Hence, the UK governments’ COVID-19 response included a national ‘lockdown’ with additional guidance for clinically vulnerable people, which included those with Type 2 diabetes mellitus (T2DM) [3]. Those considered *extremely* clinically vulnerable (2.2million people with certain single or multi-morbidities such as cancers and heart disease) were advised to ‘shield’ in their homes for 12 weeks from 22nd March 2020 and avoid all in-person contact with others even within their own household [4]. Evidence suggests that clinically vulnerable people, and particularly those required to shield, experienced stark reductions in physical activity and high levels of depression, anxiety and loneliness during the pandemic [5].

Social prescribing takes varying forms ranging from signposting to holistic support [6, 7]; however, the key component tends to be a non-medical ‘link worker’ who helps patients achieve personalised health goals by referring them into a range of non-clinical local voluntary, community and social enterprise (VCSE) sector activities, services or groups, as well as resources supplied by local government and health services. The intervention evaluated in this study predated the large-scale roll out of social prescribing in the UK by the National Health Service. The intervention had originally been commissioned by the local Clinical Commissioning Group and was managed and delivered by VCSE organisations; patients were referred by a health care professional at their GP surgery and link workers were attached to GP surgeries. Examples of referrals by link workers include for social security and housing advice, and to physical activities and social and community classes [8, 9]. Pre-pandemic, literatures have raised issues regarding inadequate training, and the capacity of link workers to support vulnerable clients with complex needs [10–12], and the effectiveness of social prescribing for improving patient outcomes in areas of high socio-economic deprivation [13]. Social prescribing is regarded as an intervention with potential to be remodelled to meet exacerbated public health challenges by supporting those most vulnerable to the impact of COVID-19 and social distancing [14, 15]. During much of 2020, health services were overwhelmed, and support offered by link workers was considered to be an important resource for vulnerable people [15].

Several qualitative studies have investigated clients’ or link workers’ experiences of social prescribing with the aim of understanding how social prescribing works and what challenges exist regarding delivery and client engagement [6, 7, 16–21]. These studies indicate that clients have positive experiences of social prescribing, with some evidence of improved wellbeing and reduced social isolation and that well-networked link workers highly skilled in empathy, listening, and who build trusting relationships with clients are essential for successful social prescribing [19, 20, 22]. However, threats to this success include high levels of staff turnover in connection with low pay and temporary contracts [23], challenges of developing relationships with primary care practitioners [24], and the capacity and sustainability of linked VCSE services [25], as identified from the perspectives of GPs [26], link workers [20] and VCSE sector actors [24]. Recent evidence from social prescribing stakeholders in Scotland during the pandemic showed an escalation of issues of limited VSCE sector capacity (including closures) and increased waiting times for mental health services [27].

Exacerbated inequalities witnessed during the COVID-19 pandemic in the UK and the unequal distribution of COVID-19 infection and mortality [28] create cause for concern amongst already vulnerable clients of social prescribing interventions. Social prescribing clients, who often have complex health conditions that significantly affect their lives, are known to experience ‘set backs’ in their health [18, 21]. It is unknown to what extent remote delivery of social prescribing service provision might affect vulnerable clients’ engagement, health and wellbeing. This study contributes to a growing body of social prescribing literature by exploring the delivery and receipt of a social prescribing intervention in a deprived area in an unprecedented context of disruption.

This study explored service providers’ accounts of how a social prescribing intervention adapted to meet clients’ needs in the first wave of the COVID-19 pandemic alongside clients’ accounts of how the intervention worked for them. This paper describes changes to the service, and explores the challenges and opportunities for the service during the first wave of the COVID-19 pandemic.

## Methods

### Context and the intervention

This study was part of a larger evaluation of a social prescribing intervention. During the time of this study (May–September 2020), clients and service providers were living under government COVID-19 restrictions, living under ‘lockdown’ from 23rd March to 12th May [29]. The study was set in an ethnically diverse urban locality (including urban fringes) in North East England. The locality is one of the 20% most deprived Local Authorities in England with higher than national average rates of premature mortality from cancer, cardiovascular, respiratory and liver disease, and starkly unequal life expectancy between more and less affluent areas [30]. The participants were either delivering or receiving the VCSE-commissioned model of social prescribing which targeted people aged 40–74 who had at least one of the following LTCs: diabetes type 1 and 2, heart failure, coronary heart disease, epilepsy, osteoporosis, asthma, and/or chronic obstructive pulmonary disease with or without anxiety and/or depression. The intervention was delivered by link workers employed by two VCSE organisations and clients could engage for up to approximately 3.5 years. Further details about the model of social prescribing is reported in the larger evaluation study protocol [31].

### Recruitment

Service provider participants were either already participating in a link worker ethnography as part of the larger social prescribing intervention evaluation [31], or were recruited specifically for interview via local connections and snowballing techniques. Client participants were those taking part in an 18-month ethnographic study exploring client experiences of the intervention [32] or recruited from a sample participating in a quantitative quality of life (EQ-5D-5L) study. The former were interviewed about their experiences of the pandemic as part of the ethnography. The latter were contacted to complete a 12-month follow-up questionnaire during the lockdown period, 90 of whom were asked if they could be contacted to participate in an additional telephone interview about their experiences during the pandemic. Forty-nine agreed to be contacted for the interview study. Thirty-six were approached but four did not respond and three declined to participate. The authors judged this a sufficient sample size as it represented a diverse demographic and range of experiences.

Participants received information leaflets and consent was recorded verbally prior to the interview due to the lockdown restrictions. Telephone interviews were recorded with permission.

### Data collection

This study is situated within the social constructionist paradigm [33] and utilised a qualitative design aligning with the study aims of exploring in-depth service providers’ and clients’ experiences of delivering and engaging with social prescribing during the pandemic. Semi-structured interviews were chosen to elicit accounts of social prescribing rather than a direct representation of events and enabled unanticipated themes to emerge [34]. Service providers and clients were interviewed by experienced anthropologists (SLM and TP) and sociologists (SM, JJ and KG) via telephone (due to COVID-19 restrictions) between May 1st and July 13th 2020, with one additional interview with a service provider conducted in September 2020. The client interview topic guide covered experiences of ill-health; impacts of COVID-19 on interviewees and their households and, their LTCs; support needed or received including social prescribing; and, feelings about life during and after lockdown. Demographic data were collected on age, gender, ethnicity, employment, education, household income, housing tenure/type/composition and availability of private outside space. Service provider interviews followed topic guides which were tailored to each individual but included questions regarding the organisation’s philosophy, adaptations since March 2020, and organisational and individual challenges and opportunities. Demographic data were not collected for service providers because of the risk of identifying individuals.

### Data management and analysis

Audio recorded interview data were transcribed verbatim by a University-approved professional transcription company. The transcripts were checked by the research team, anonymised and then read and re-read closely. A priori and inductive coding frameworks were developed by JMW, SLM, BG and KG and discussed with other authors before applying to either client or service provider transcripts. NVIVO 12 was used to assist with data management: the client and service provider data were managed separately due to the differences in topics covered in interviews. We followed a thematic approach to analysis [34, 35] to identify patterns relevant to the research questions with line-by-line coding used to identify descriptive categories. Rigour was ensured by checking independently coded transcripts. The constant comparison method was used to develop conceptual themes, some of which spanned the data sets. Client case summaries were used to trace their experiences of the intervention to their general experiences of the pandemic [36]. Data were analysed as a ‘snapshot’ in time during the changing circumstances of the pandemic and report accounts of the service changes that inevitably altered over time as the pandemic continued into the later months of 2020. Authors met regularly to discuss the emerging themes and developed the final analysis iteratively.

Ethical approval was gained from the Durham University Ethics Committee, all methods were conducted in accordance with the Declaration of Helsinki (ethical principles for medical research) and all participant quotes are anonymised in this paper using participant codes and pseudonyms.

## Findings

### Participant characteristics

The social prescribing service providers comprised eight service managerial staff and five link workers and senior link workers across two provider organisations. The client sample comprised 44 individuals (see Table 1). Clients were mostly aged over 50 years, over one third were retired and nearly two thirds were homeowners. Over half of the sample claimed some form of social security benefit, many of which were related to their health status. Nearly one third were unemployed, often due to ill health, and nearly two thirds reported annual household incomes of less than £20,000. All participants had been with the intervention for 12 months or more and, as such, many may had been moved to less frequent link-worker contact.. Nine clients reported having a single LTC. The remaining 35 had multiple conditions, which most commonly comprised T2DM, hypertension, asthma, chronic obstructive pulmonary disorder (COPD), fibromyalgia, depression and anxiety. Some had a more complex mix of LTCs including multiple sclerosis, vascular disease, heart failure, epilepsy and cancers. Over half of the sample experienced struggles and difficulties with their health and wellbeing during the first wave of the pandemic, and some experienced significant disruptions to their employment and finances.

**Table 1:** Participant demographics

	N
**Gender**	
Male	19
Female	25
**Age**	
40–49	6
50–59	11
60–69	17
70+	10
**Ethnicity**^a^	
White British	38
British Bangladeshi/Pakistani/ Indian	6
**Income**	
< 10 K	13
10-20 K	14
21-30 K	6
31-40 K	3
> 40 K	4
**Employment status**	
Prefer not to say	4
Full-time (FT) employment	4
Part-time (PT) employment	8
Furloughed	2
Unemployed	13
Retired	17
**Benefits claimed**^b^	
None	20
Health-related benefits	18
Means-tested benefits	6
**Number of LTCs**^c^	
1	9
2 or more	35
**Household Structure**	
Lives alone	12
Lives with partner	17
Lives with family < 18 yrs	10
Lives with family > 18 yrs	4
Multigenerational household	1
**Housing Status**	
Owned	26
Rental (Private or social housing)	17
Other	1
**IMD quintile of home address**	
1 (least deprived)	6
2	4
3	9
4	5
5 (most deprived)	20

^a^The ethnic diversity of the sample is similar to the population of Newcastle-upon-Tyne, with 88% of the population being White British and Asian/British Asian ethnic groups comprising the majority of the minority ethnic groups (ONS, 2011)

^b^Means-tested benefits include Universal Credit and Working Tax Credits which are available to people on a low income; and Income Support which is available for people who are out of work or unable to work. Health-related benefits included PIP (Personal Independence Payment), DLA (Disability Living Allowance) and ESA (Employment Support Allowance) which are available for people who have a disability or health condition that affects how much they can work. We also include Carers and Attendance Allowance in this category, and Universal Credit LCW (Limited Capacity for Work), which is an additional element of UC for people who have limited capacity to work due to a health condition or disability

^c^LTCs were self-reported by participants in interviews

### Service providers

This section reports accounts of how the intervention was adapted during the first months of the pandemic; the innovations, opportunities and challenges that link workers faced in remote delivery; and how clients were perceived to engage with the intervention during this time. Supporting quotes for each theme are referred to in the text and located in Table 2 and are linked to participants by anonymous codes: [Manager/Senior Link worker (SLW)/Link worker (LW)][Interviewee number]_[Interview date].

**Table 2:** Service Provider interview quote examples

**Intervention adaptations to COVID-19 crisis: March–June 2020**	
*(LWC_31_06/20)*	*… We’ve been given shielding lists by the practices, so the people who are vulnerable, we’re checking on them, but that is not our normal conversations again. That’s just like, “Do you have food and medicine?” if you know the guidelines, stuff like that … checking that people are okay and they’ve got access to food and medicine, that they’re coping with it mentally and they know what they should be doing.*
*(Manager_3_06/20)*	*The type of support that we have been providing has changed slightly as well, in the early days. So, people were very worried and concerned, naturally, about COVID and what the likely impact that would potentially have on themselves and their health. They needed information around what they could and what they couldn’t do. So, it was just really to help them understand what the government was saying. So, a lot of time, initially I think, was supporting people around understanding of what the lockdown restrictions meant for them and finding out what support they needed. Because obviously, a lot of people don’t have family or friends and if they are shielding or isolating, they can’t go to the shops to get their shopping and stuff like that.*
*(Manager_4_05/20)*	*So, if you have improved your condition by getting out a bit more and feeling more hopeful and enlightened by that, and now you have been told you can’t go out, then there will be that bit- It is much easier to do something the first time than if you have gained some success and then slipped back. So, it is that bit of just trying to help people wherever possible not to lose the ground that they have gained.*
*(Manager_2_06/20)*	*I think the priorities initially were right, you know, checking in on the people in our current caseloads, making sure they had contact, and whether there was anything that they needed- that sort of immediate support-, or they still wanted to engage with ongoing support … Whilst obviously it has chucked everything up in the air for everybody, you know, still using our skills and our knowledge to be able to support people to manage this as best they can, and identify if there are goals, although they will be reframed, to still be able to work towards that even in the current climate.*
*(SLW_32_06/20)* *(Manager_8_09/20)*	*The nature of our role is that we are very flexible and we maintain contact. It is very much a contact role and a communication role. I think that is important for people who have been self-isolating and haven’t left their homes. Some people: maybe the furthest they have gone is the garden. It is reassuring to know that you are ringing, they can contact you or if they are having problems with things, that you can ask the right questions to the right people and you can feed back information. Even if you haven’t got the information at the time, to be able to tell them that you are still waiting for information, but you have asked the question. I think that gives reassurance at a very difficult time. I think that is definitely a strength* *… there was a sense that people were like, “Why would I discharge them at a time when everything in their life is up in the air?” So I think there was a sense that they were, kind of, keeping things open for a little while. I don’t think that’s the case anymore [in September]. I think things feel more stabilised*
*(Manager_2_06/20)*	*I think there was getting that balance between, “Here are the people that I know that I’ve been working with that I would identify as a priority.” But actually recognising that it was important for us to try and check-in with as many people, if not everybody, on our caseload to try and see what the impact of this situation was*.
*(Manager_1_06/20)*	*So we also had discussions with them about understanding on the caseload, people that actually probably didn’t need our support. Just to be able to discharge, for want of a better word, people that probably were coming to the end of their intervention with ourselves so we could really focus on those that really did need our support.*
*(LW_9_06/20)*	*But because of the current situation, I haven’t closed them or anything, because of the current situation.*
**Link worker innovations and challenges with remote delivery to clients**	
*(LW_9_06/20)*	*if you’re not digitally connected there’s an obvious gap for a lot of our clients’*
*(LW_33_06/20)*	*Some of our clients aren’t particularly tech-savvy. Some are on the internet and doing FaceTime with grandchildren and all that kind of thing, but I do have … Fairly recently … I’m finding this a challenge and I’m finding it difficult to know how to overcome really. I do have a couple of clients that are unable to read or write. Ordinarily, where I’d be sending an email with some links to websites or something like that for information during this time or even posting out something, printing something off my own printer, I don’t have that option with these two individuals*
*(LW_31_06/20)* *(LW_31_06/20)*	*The transition to phone calls has been difficult, because even though we do that anyway, when we first do a [assessment] with someone, the face-to-face of that is so important because you’re introducing yourself, what the service is. You need to build that rapport, otherwise people aren’t going to open up to you. So, introducing that over the phone has been quite hard. Then everything has changed, so you can’t see what people are doing body-language wise, face wise. We’re told to hold silences, but that is very different over the phone* *“things aren’t open, so things that I might like to signpost people to, I can’t.”*
**Perceptions of client engagement during remote delivery and a crisis**	
*(SLW_32_06/20)*	*The people who I have spoken to don’t express an interest in anything like that [online activities]. I mean, a good example would be a client who was attending Slimming World. He mentioned about the Zoom facility that Slimming World that have and he said it didn’t work for him. It wasn’t the same as actually going to a group, seeing people and talking to them. This 2D representation, if you like, wasn’t the same as having that actual social contact.*
*(LW_33_06/20)*	*Some clients are like, “Oh, I prefer face-to-face because then I can see your facial reactions and your body language and all the rest of it.” It’s the same, I guess, for us, because we can’t really see how things land. If we’re saying something, a lot of the communication cues are visual rather than verbal.*
*(LW_9_06/20)*	*People who haven’t answered the phone are now answering the phone and they’re wanting to chat. Now that is brilliant, because a lot of people … who wouldn’t have wanted to come to the doctor’s surgery [to visit the link worker], or find it difficult … they will happily chat on the phone for half an hour, an hour, and they’re really engaging at quite a good level with that, they’re someone who is DNA [did not attend], coming to the doctor’s surgery before. And when I’m now chatting to them, it’s obvious that actually to physically come to the doctor’s surgery is difficult.*
*(LW_9_06/20)*	*nearly everyone picks up the phone immediately, and they want to talk to you, they haven’t got other stuff going on. So that’s been amazing. (Laughter) Partly because a little bit of their worries and stuff- They are quite open to ideas to benefit their health, because actually they’re a bit frightened. … I think the COVID thing has opened up people to a whole load of different new ideas. I kind of sense that a bit, that some people are just thinking, “Oh, I’ll give that a go.” It’s like opened up a different intensity about their health almost, that they’re now thinking that there might be other ways of doing things, and stuff*
*(SLW_32_06/20)*	*We are very social and there is a big social element, I believe, in motivation. For example, if somebody wanted to talk about smoking cessation, we would discuss realistic approaches and, maybe, reduction with a view to them moving on to cessation. I find that that can be quite difficult because people may be motivated to want to change, but not having that contact as well... That contact can be reassuring. “Oh, I can go and see [Link worker] at the GP.” They can book the appointment and come in and see me, have a chat about it and have a real face-to-face conversation as opposed to over the phone.*
*(LW_31_06/20)*	*they [clients] don’t really care about behaviour change at the minute … I think people just don’t really want the conversation that we want. They’re happy to talk with someone because they’re so bored-“I think it is just trying to stay on track of what we actually are, which is a behaviour change service. …. So, even if we’re not doing the conversations like I said, not all focused on behaviour change, we’re still speaking to someone, listening to them and if they do desperately need anything, we can signpost them to that. That is the main job of social prescribing, doing the signposting.*

#### Intervention adaptations to the COVID-19 crisis: march–June 2020

A few days prior to the UK national lockdown on March 23rd 2020, service providers switched intervention delivery to a remote working system. Link workers reported communicating with clients by telephone appointments whilst working from home. In addition to existing clients, link workers began working with people classed as ‘vulnerable’ on GP surgery and city council lists to adjust the service to *“unmet need out there in the community and really vulnerable people”* (Manager_8_09/20) who did not meet the intervention’s original eligibility criteria. With vulnerable list clients, link workers reported focusing on immediate help and referrals to other services such as food and medicine supply deliveries, checking people understood government COVID-19 guidelines, and that people were coping. This was described as *“not our normal conversations”* (LW_31_ 06/20). One link worker reported that approximately 15–20% of vulnerable list patients needed additional support, whereas most required a check-in phone call only. The intervention’s rapid adaptation to assisting with vulnerable patients in a time of crisis, provided an opportunity to strengthen relationships with primary care. It was hoped GP practices would “see how important social prescribing is” and utilise the service more (Manager_8_09/20).

Link workers and managers reported a change in the intervention for existing clients too. Conversations were said to have initially altered in content as they provided advice, information and assistance surrounding COVID-19. Managers explained COVID-19 advice and support was a priority in the early months of the pandemic, but also suggested there were attempts to balance this short-term COVID-19 response alongside reframing behaviour change goals and supporting people not to ‘relapse’ with their condition management (e.g. Manager_2).

It was commonly recognised that the pandemic could have altered existing clients’ lives dramatically and that some who were doing well prior to lockdown may subsequently be struggling. Link workers said that maintaining contact with clients was key during the lockdown and the use of telephone calls meant clients were often easier to access than before lockdown (e.g. SLW_32). Retaining contact with clients was expressed as a priority. As such, managers and link workers reported balancing contacting clients with known high levels of need with an aim of contacting entire current caseloads and, in some cases, recontacting clients who had been previously discharged (e.g. Manager_2). Differences in approaches were evident, with some link workers and managers reporting choosing not to discharge clients during the pandemic, whereas other link workers were encouraged to close files on clients considered no longer in need of assistance (e.g. Manager_1).

#### Link worker innovations and challenges with remote service delivery

Some link workers were “*creative*” and innovative in how they supported clients during remote delivery. Some said they were responding to client need, supporting and advocating for clients more. Others explained how they were reproducing in-person support remotely, including one who was trying breathing activities remotely with clients with COPD, as a substitute for attending in-person classes.

Link workers reported challenges in engaging with and supporting digitally excluded clients during the pandemic (e.g. LW_33). Some attempted to adapt their delivery to digitally excluded clients by posting hard copies of information, often at their own expense. However, this adaptation was inaccessible for illiterate clients.

In addition to difficulties with communicating information, many link workers were unclear about which linked services were running remotely, and expressed difficulties in fulfilling their roles when the usual link services were unavailable. Some link workers also expressed difficulties with building rapport over the phone if they had not met the client in-person previously. One link worker (e.g. LW_31) expressed how it was challenging to put her conversational and therapeutic skills into practice remotely and that remote delivery from the outset of client journeys (never meeting a client in-person) had implications for how she interacted and engaged with clients.

#### Link workers’ perceptions of client engagement during remote delivery

Remote working brought perceived differences in clients’ interactions and engagement with the service. Some link workers said there was an overall increase in anxiety in clients and that most were pleased to have ‘check-in’ telephone calls. Some link workers said many of their clients were not interested in online service provision (e.g. SLW_32). Although some said their clients preferred face-to-face interactions, one link worker (e.g. LW_9) explained that some clients were more engaged than previously due to remote appointments, and suggested that this change made the service more person-centred, with clients being more open, inventive, and adaptive during the lockdown due to re-evaluating their health and vulnerabilities.

Some link workers and managers suggested that the intervention was primarily concerned with behaviour change and that the lockdown and remote interactions were affecting some clients’ motivation to engage. One link worker (e.g. SLW_32) suggested that in-person social interactions could be more helpful for client behaviour change than the remote conversations they were now conducting, whilst others said clients were not thinking about long-term change in the current climate (e.g. LW_31).

### Clients

This section details clients’ experiences of the intervention and draws attention to contrasting findings between the client and service provider data. We first discuss the variety of client experiences before describing how some experienced a sense of being ‘on hold’ or losing contact with social prescribing, which led to ‘set backs’ in health. Client quotes feature in Table 3 and are contextualised using the following codes: [pseudonym name*_*age group*_*employment status*_*IMD decile*_*household structure*_*number of conditions (1 or 2+)_interview date (e.g. 01/01/21)].

**Table 3:** Client interview quote examples

***Varied contact with link workers***	
Jude_60–69_Unemployed_IMD 5_lives alone_2 + _09/06/20	*No, the last time I heard from her was the beginning of the year, it was either January or February. It was before my tribunal, because she said, “I wish you luck,” and that. And then the lockdown happened just after that …. No. They’ll not be working, will they?... I’ve got her phone number, if I need to ring … You know, if I need to ring them, I could ring. But I haven’t. Well, like I say, everybody is on lockdown, so … There will be a lot of people that aren’t back at work yet, you know. I didn’t expect anything anyway at this time.*
Jessica_40-45_full-time employment_ IMD 5_lives with family-2 + _18/05/20	*[Name of link worker (LW)] telephoned me and I had a good conversation with LW. And then, as I say, she emailed me some resources and things over, as well, so that was really helpful … Just regarding my diabetes and some bereavement information for [daughter], as well …*. Y*es. We arranged to speak again … we said we’d leave it six weeks from our conversation, so it will be June, yes …. We’ve set a couple of targets, and I thought that gives me time to get my head around it and get going with it. (Laughter) … I’ve discussed actually with [LW] that, when we did the outcome star and things, we both said that- I think [husband] and I support each other quite well, and I think that really helps, I do feel like I have a lot of support from my husband.*
Eddie_50–59_unemployed_IMD 1_lives alone_2 + _06/07/20	*He called, [LW], a couple of weeks ago I think …*. [*intervention], yes. Just a catch up thing, and he got this organisation to come down and drop off a little food parcel thing …. that’s the only time I’ve heard from them … He called me out of the blue. I hadn’t heard from him for a little while before that … .he delivered three of them [foodbank vouchers at the start of lockdown] … I used two of them. I wasn’t up for going for the next one.*
Gill_60–69_unemployed-IMD 1_lives alone_2 + _shielding_06/07/20	*I thought that she was the kind of person that I could actually talk to because I was really, really nervous on the way there because- like I have said, I don’t care much for seeing people who I don’t particularly know very well. But, I knew I had to go and then I found her manner was just exactly what I needed. She wasn’t condescending or she wasn’t pushing me to do things that I really didn’t want to do at the time, and she has been quite a valuable person to me, ringing up and seeing how I am and things …. Well, it is somebody I can talk to who knows exactly what I have gone through, you know …. When she rings up, I always feel a lot better after I have been speaking to her … But especially at the time where I was going through the devastation of losing Jim and everything, oh, she was just brilliant, you know. She has given me so much encouragement and she tried to get me to see a positive side of things, as well, you know*
***‘Just waiting’: social prescribing ‘on hold’***	
Derrick_60–69_Employed part-time_IMD 1_lives alone_2 + _18/06/20	*I’ve had a few interviews [with the LW] and stuff and they said they’re going to try and get me into a gymnasium. But with everything closing down and stuff- Swimming and stuff for my legs … So yes, I’m just waiting for that now … When the virus started, I’ve had a couple of phone calls off a lad, a man, but I don’t know who he is. But he was asking how I was and stuff like that, and how I’m coping. And they’re going to keep in touch … But with this virus, there’s not much they can do. Because their hands are tied with what they actually can do. Because I can’t actually go and physically see them one-on-one … if I see them one-on-one, it’s a lot better, I’ll get my point across a lot more, because I’m actually talking to another person, than being on the phone. Like telling them more, if you know what I mean, like face-to-face … So I hope, once this virus is over, I actually go down there and have a one-on-one interview with them and discuss what I’ve been doing through this virus and how my legs have been and how I’ve coped at work and stuff like that, just see what can be done.*
John_50–59_Employed full-time (pre-lockdown)_IMD 3_lives with partner_2 + _shielding_28/05/20	*Since this started [COVID-19], I’ve put the weight [on] … There is just no exercise whatsoever.* *It [the intervention] was great. The guy I was talking to was brilliant. [link worker name]. I’ve forgotten his second name. Everything started working. I gave up smoking and I was losing weight and then COVID-19 entered the scene and just ruined everything … .. Just the guy I was talking to was brilliant and that. His opinion and perspective …*. H*e told me that I’m being too hard on myself. Yes, it was really good …. He [another link worker] phoned up a few weeks ago just to see how I was doing. He was alright. It’s a different guy. It changed. The last time I was there it got changed. He′s not as good as the first one, but he’s alright …*. J*ust [spoke about] the same sorts of things. Smoking, putting weight on, state of mind …* *For me personally, no [not keen on online exercise classes]. I need some motivation. I need to be at the gym with somebody telling me what to do.*
***Losing contact with social prescribing: Struggling and going ‘back to square one’***	
Reena_60–69_Employed Part-time (pre-lockdown)_IMD 2_lives alone_2 + _shielding_18/05/20	*Now I’m right back to square one again …. You just get forgotten, don’t you? You just feel like you just disappear. That’s how I feel. I’ve just disappeared.* *I’m not very good at … If she’s not expecting me to call, I don’t know. I don’t know her … I knew the old one. I knew the old one quite well. I don’t know her.* *I don’t know how it can be, because you can’t really do anything or go anywhere. I think this is just how it’s got to be, in a way …. You’ve just got to put up with it I think, and get on with it … .. at the moment I don’t know what they [intervention] can do really, apart from just talk to me maybe.*

#### Varied contact with link workers: from ‘check-ins’ to valued support

Clients’ accounts suggested that the service providers’ aims to contact all clients were patchily achieved. Some recalled regular or irregular contact with their link workers during lockdown. However, most participants had not heard from their link worker during lockdown and a few did not recall the intervention or a link worker. Some were not expecting a call due to an understanding they had been ‘signed off’ or because they knew their next scheduled call was at a certain point in the future. However, others were surprised by the lack of contact or were unaware that link workers were working during lockdown (e.g. Jude).

Some people who received contact said they did not need support. These individuals commonly experienced a “convivial chat” or check-in as an enjoyable interaction. Others received more valuable support, such as one participant (Jessica) who had received bereavement support from her link worker prior to lockdown. She explained how the link worker had called during lockdown and then sent her valuable resources over email for managing her condition and other issues. Jessica was very conscious of her own and her family’s health, aimed to ensure all family members were engaging in some form of physical activity during the restrictions, and took additional protective strategies to reduce their risk of COVID-19 infection. Jessica had anxieties around COVID-19 and her family had had to adapt their working patterns to manage. Nonetheless, aside from the pandemic, they were living in a relatively stable context thus enabling Jessica to prioritise her health conditions.

A few participants who were living in more challenging circumstances received useful support from the intervention during the first lockdown. For instance, one participant (e.g. Eddie) explained how he received unexpected assistance with the delivery of a food parcel and foodbank vouchers. Another client (e.g. Gill) received consistent social and emotional support from her link worker over a course of several months prior to and during the pandemic. The link worker-client relationship was central to the success of this support. Gill, who had multiple complex health issues, severe depression and a limited social network, said she had difficulty trusting new people. However, when she first met her link worker she found her easy to talk to and non-judgemental. She explained how during the course of the pandemic when her partner passed away, the link worker became even more important in helping her with this traumatic bereavement and providing access to counselling. Gill had waited many months for counselling, and after this had ceased (after 6 sessions which were “only just touching the surface” of Gill’s problems), Gill reported that the link worker was trying to source other counselling for her. At the time of the interview in early July 2020, the link worker and Gill were speaking every other week, and Gill knew when to expect her calls. The continued link worker relationship was key for Gill in a time of traumatic disruption, and the intervention she received was person-centred, holistic and consistent.

#### ‘Just waiting’: social prescribing ‘on hold’

Some clients felt their journeys with the intervention were “on hold” and they were “just waiting” as they could not physically see their link worker or be linked to services they required. In this temporary liminal space, some clients’ abilities to take action regarding their health, or for the intervention to help them with self-management, were limited. For instance, Derrick (See Table 3) said the intervention kept in touch with him during lockdown, asking whether he was following dietary advice and keeping well. However, his link-worker had changed, and the intervention’s role and his ability to do anything during this period were limited. Derrick viewed the intervention as temporarily suspended but appreciated them ‘checking in’. Similar to some of the link workers’ difficulties building rapport over the phone, Derrick felt he could communicate his experiences and goals better in person. During the lockdown, plans made for a gym referral and help with housing were “on hold” (a term used by others in relation to many aspects on their lives and healthcare) and he described himself as “just waiting”. Derrick explained he was becoming depressed and had been “comfort eating” high sugar food despite knowing it would adversely affect his T2DM. However, his poor living conditions and social isolation hindered positive practices and uptake of the “good advice” about diet he said he had received from the intervention. In contrast to the perspectives of some link workers, clients such as Derrick appeared to be thinking long-term about their health but the circumstances and being ‘on hold’ with social prescribing meant they remained in a present where it was impossible to act on any long-term health goals.

The cessation of formal in-person groups and gym services facilitated through social prescribing was damaging for several clients who had made significant progress with their health prior to lockdown. For example, despite contact from a link worker, John, who was required to shield due to his health conditions, expressed feeling *“back to square one”* with his condition management, in part because of the temporary closure of gym services (See Table 3). Online exercise provision did not suit John, who credited his pre-pandemic success in increasing his physical activity and stopping smoking with the motivation provided by his previous link worker and the trainer at the gym he was attending.

Some less digitally literate or less digitally-connected clients reported that linked services had attempted to engage with them. For example, Christine (60–69–Unemployed–IMD 1–lives alone–2+), who lacked internet access, could not join in with her art class on Zoom. Instead, she received art materials from the class in the post, which she said she appreciated. However, she continued to miss the group and the staff member who often helped her navigate the benefit system.

#### Losing contact with social prescribing: struggling and going ‘back to square one’

Some participants who were struggling with condition management and social isolation did not have any contact from their link worker during the lockdown. For example, when Reena (See Table 3) was first referred she explained that her link worker had been “really good” in providing “access and information on lots of activities” such as swimming, circuit classes, tai chi classes, and a walking group. This support enabled her to make dramatic improvements to her health and wellbeing. However, support “dwindled off a bit” when she had a change of link worker before the pandemic and she had not heard from the service during the pandemic. Reena expressed apprehension about contacting the link worker because she did not know them or what formal support they could offer. She and others said they did not need food or medicines delivered as they were receiving online deliveries or had spouse/family support for errands. The limited client-link worker relationship and lack of contact meant that Reena and some other clients’ changing circumstances and struggles during the pandemic were not identified, and thus support was not provided.

## Discussion

This paper explored accounts of how a social prescribing service changed in response to the first wave of the COVID-19 pandemic, including the challenges and opportunities from the service provider perspective and how clients experienced the intervention. We found that the service adapted in order to respond to the needs of vulnerable people. The content of the intervention reportedly changed in the early months of the pandemic. Similar to recommendations set out for community health workers (a form of link worker) elsewhere, often in low-income and middle-income countries [37], the intervention aimed to integrate with existing local infrastructures to support the most vulnerable. Service providers set out to assist with immediate needs (e.g. food parcels, medicine arrangements) and provide support around COVID-19 guidelines and anxieties in both the existing client base and a wider group of vulnerable people. Similar changes were highlighted by stakeholders in social prescribing schemes serving both rural and urban areas of Scotland [27]. Existing clients had varied experiences of the intervention. Some received ‘check-in’ calls, others experienced more in-depth and valued assistance with self-management or social support, while others reported not hearing from their link workers. Some link workers reported innovations developed to support people when linked services were unavailable and when clients were no longer able cope in ways they had done previously. Nevertheless, some clients were struggling with their health and lacked any formal support. Others who were in contact with link workers still felt their journeys were ‘on hold’ or were ‘back to square one’ due to the disruption of linked services such as gyms, walking groups, and social groups. Amongst the multiple challenges of operating a social prescribing service during the COVID-19 pandemic, our findings illuminate the vulnerability of social prescribing when VCSE sector and linked services are not accessible, an issue reported widely in the social prescribing literatures prior to and during the pandemic, for link workers situated both in primary care networks and in VCSE organisations [19, 23, 27].

The COVID-19 pandemic created opportunities and challenges for clients’ engagement with social prescribing. Service providers reported better access to clients via telephone in the first months of remote delivery. However, some link workers expressed difficulties engaging clients who were digitally excluded or illiterate via remote methods, and some clients reported that services, including online exercise or art classes, were inaccessible or did not work for them. Evidence suggests that digital exclusion has coupled with social exclusion during the pandemic, particularly in older adults with multi-morbidities and functional impairments [38]. The increase in digital inequities in access to healthcare during the COVID-19 pandemic will require “intensive, long-term support networks” to address digital exclusion and help people develop the skills and access to technology needed for digital inclusion [39]. Providing additional support to enable digital inclusion will require extra capacity from link workers who already often experience high caseloads [20]. Without additional support, digitally excluded clients will struggle to engage with remotely and/or digitally delivered social prescribing, which will likely continue post-pandemic [40].

For one client (Gill), the intervention became a central resource for support during a traumatic period made worse by COVID-19. This experience acts as an example of how social prescribing can work successfully in a person-centred way for vulnerable clients at times of crisis. However, our study demonstrates that this potential was contingent upon a consistent trusting link worker-client relationship, link worker capacity, and skills in dealing with complex physical and mental health issues, trauma and disruption. A Research-in-Residence study of the implementation of social prescribing in South West England sites found that link worker skills, knowledge and background affected their confidence and innovation in responding to new challenges [22]. High staff turnover and high link worker caseloads have been noted in previous studies [12] and are recognised as issues negatively impacting on the potential for providing personalised care and supporting clients to achieve long-term health and condition management goals [21]. Similarly, in our study, many clients recalled a change in link worker, which sometimes had negative consequences, including demotivation and isolation, and played out in a particularly damaging manner for some. The contrasting client examples presented in our analysis show that link workers’ abilities to detect changes in people’s lives in the pandemic were paramount to the client’s experience of and engagement with social prescribing.

To our knowledge this is the first substantial qualitative study exploring social prescribing during the COVID-19 pandemic from both the client and service provider perspective. However, it is not without limitations. Our sample of link workers was small and data are time limited to the first months of the pandemic. Although the intervention featuring in this study is unique in its funding structure and target population, findings are indicative of wider issues in social prescribing schemes.

## Conclusions

The social prescribing intervention in this study set out to respond sensitively to the needs of local people during the first few months of the pandemic and deliver much-needed support to many. However, limitations likely created by high caseloads and staff turnover meant that while some clients experienced substantial help from their link worker, others, including those living with more challenging circumstances and health issues, did not. Our findings suggest that if social prescribing is to succeed in alleviating social isolation connected with COVID-19 [14], link working needs to be consistent, frequent, and services need to be made accessible to digitally excluded groups.

## Data Availability

The datasets generated and/or analysed during the current study are not publicly available due to their containing information that could compromise the privacy of research participants but are available from the corresponding author on reasonable request.

## Abbreviations

COPD: Chronic obstructive pulmonary disorder
LTCs: Long-term conditions
LW: Link worker
SLW: Senior Link worker
T2DM: Type 2 Diabetes mellitus
VCSE: Voluntary, community and social enterprise
